# Risk Factors for Obstructive Sleep Apnea Are Prevalent in People with Psychosis and Correlate with Impaired Social Functioning and Poor Physical Health

**DOI:** 10.3389/fpsyt.2016.00139

**Published:** 2016-08-31

**Authors:** Dennis Liu, Hannah Myles, Debra L. Foley, Gerald F. Watts, Vera A. Morgan, David Castle, Anna Waterreus, Andrew Mackinnon, Cherrie Ann Galletly

**Affiliations:** ^1^Discipline of Psychiatry, School of Medicine, Adelaide University, Adelaide, SA, Australia; ^2^Northern Adelaide Local Health Network, Adelaide, SA, Australia; ^3^Country Health, Adelaide, SA, Australia; ^4^Centre for Epidemiology and Biostatistics, Melbourne School of Population and Global Health, The University of Melbourne, Melbourne, VIC, Australia; ^5^Lipid Disorders Clinic, Department of Cardiology, Royal Perth Hospital, School of Medicine and Pharmacology, The University of Western Australia, Perth, WA, Australia; ^6^Neuropsychiatric Epidemiology Research Unit, School of Psychiatry and Clinical Neurosciences, The University of Western Australia, Perth, WA, Australia; ^7^Department of Psychiatry, The University of Melbourne, Melbourne, VIC, Australia; ^8^St. Vincent’s Mental Health, Melbourne, VIC, Australia; ^9^Orygen Youth Health Research Centre, University of Melbourne, Melbourne, VIC, Australia; ^10^Centre for Youth Mental Health, University of Melbourne, Melbourne, VIC, Australia; ^11^Ramsay Health Care (SA) Mental Health, Adelaide, SA, Australia

**Keywords:** risk factors, obstructive sleep apnea, psychosis, social functioning, physical health

## Abstract

**Background:**

Obstructive sleep apnea (OSA) in the general community is associated with obesity, smoking, alcohol, and sedative medication use and contributes to depressed mood, daytime sedation, and sudden cardiovascular deaths. Poor cardiovascular health, impaired social functioning, and negative and cognitive symptoms are also among the common clinical features of psychotic disorders. People with psychosis have higher rates of sleep disturbance; however, OSA has not been extensively investigated in this population.

**Aims:**

This study aimed to determine the prevalence of OSA and general sleep disruption symptoms in a representative Australian sample of people with psychosis. We investigated the prevalence of potential risk factors for OSA, including obesity, psychotropic medications, and substance abuse in this population. Finally, we evaluated associations between symptoms of OSA, symptoms of general sleep disruption, and various clinical features in people with psychosis.

**Methods:**

Participants took part in the Second National Australian Survey of Psychosis, a population-based survey of Australians with a psychotic disorder aged 18–64 years. Symptoms associated with OSA (snoring and breathing pauses during sleep) in the past year were assessed using questions from the University of Maryland Medical Centre Questionnaire and symptoms associated with general sleep disruption in the past week using the Assessment of Quality of Life Questionnaire. Data collected included psychiatric diagnosis and symptoms, education, employment, medications, smoking status, physical activity, drug and alcohol use, and cognitive function. Physical health measures included body mass index, waist circumference, blood pressure, fasting blood glucose, and lipids.

**Results:**

Snoring was reported by 41.9%; 7% stating they frequently stopped breathing (pauses) during sleep. Univariate logistic regressions show OSA symptoms (pauses and snoring) were associated with older age, female gender, lower levels of social participation or employment, cardiovascular risk factors, sedentary lifestyle, and poorer quality of life, while symptoms of general sleep disruption were more likely in people with depressive symptoms.

**Conclusion:**

Australians with psychosis have high levels of sleep disturbance, including OSA. OSA symptoms were associated with cardiovascular disease risk factors, reduced social participation and employment, and poorer quality of life. Whether correction of OSA can improve these factors in people with psychosis remains to be determined.

## Introduction

Obstructive sleep apnea (OSA) affects 2–4% of the general adult population (1). OSA is characterized by repeated pharyngeal obstructions during sleep, resulting in airflow cessation (apnea) or reduction (hypopnea), frequent disruption of sleep, and hypoxic episodes. While obesity is the most significant risk factor, other risk factors, including smoking and use of sedative medications, have been found to play a significant role in OSA (2–4). OSA is associated with poor cardiometabolic outcome (5), increased risk of depression and anxiety, and impaired neurocognitive function (6).

People living with psychosis experience poor physical health and shortened life expectancy, with high prevalence of cardiometabolic risk factors, including obesity, hypertension, and diabetes (7, 8); impaired social and occupational function (9–11); and cognitive impairment (11, 12). Although OSA and psychotic disorders have many risk factors and poor clinical outcomes in common, few studies have focused on OSA in people with psychosis (13, 14). Several small studies have found a high prevalence of OSA in people with schizophrenia (15–18). High rates of obesity and smoking and frequent use of sedative medications observed in people with psychotic illnesses may contribute to the development of OSA. Alternatively, the pathological sequelae of OSA, including repeated episodes of hypoxia, sleep fragmentation, and oxidative stress, may precipitate or perpetuate psychopathological symptoms, metabolic disturbance, and impairment of neurocognitive and social functions in people with psychotic illness.

### Aims

Our primary aim was to examine the prevalence of OSA symptoms in a large representative sample of people with psychosis. Secondary aims included exploring the associations between OSA symptoms and variety of clinical outcomes, including physical health, risk factors for cardiovascular diseases, quality of life, social functioning, cognitive functioning, vocational engagement, and psychiatric symptoms. Finally, we examined the association between OSA symptoms in this population and known risk factors for OSA (e.g., obesity, alcohol and tobacco consumption, and sedative medication).

Our hypothesis was that clinical symptoms of OSA would be highly prevalent in people with psychosis and that clinical symptoms of OSA would correlate with increased cardiometabolic risk, poor cognitive and social functioning, and poor quality of life. Clinical symptoms of OSA would be associated with obesity, substance abuse, and the use of antipsychotic medications in a representative sample of people with psychotic disorders.

## Materials and Methods

### Sample

The 2010 Survey of High Impact Psychosis (SHIP) was the second Australian National survey of psychotic disorders. The survey catchment covered a population of 1.5 million people aged 18–64 years, approximately 10% of the Australian population in this age group. A two-phase design was used. In Phase 1, screening for psychosis took place in public mental health services and in non-governmental organizations supporting people with a mental illness. In Phase 2, people who screened positive for psychosis in Phase 1 were randomly selected and stratified by age group (18–34 years and 35–64 years) for interview and assessment. This process identified 7955 people who were screened positive for psychosis and eligible for interview. Potential participants were randomly selected and approached for participation in the study; 1825 participants who screened positive for psychosis were included and interviewed in Phase 2 of the study. The study was approved by the human research ethics committees at each of the seven study sites, and all participants provided written- informed consent. Full details of the survey methodology are described elsewhere (10, 12).

### Demographics and Social Participation

Gender, age, marital status, formal study educational level, and current employment were recorded. Item to assess the participant’s involvement in meaningful activity was extracted from the main interview schedule to assess: 0 = employed in any job in last 12 months; 1 = home duties/caring for own children; 2 = caring for relatives; 3 = retired; 4 = volunteer/unpaid work; 5 = student; and 6 = no formal activity. Participants were divided into two groups: no formal activity and others. Diagnostic assessment was based on a semi-structured clinical research interview, the Diagnostic Interview for Psychosis (DIP) (19). Diagnoses were made according to the ICD-10 classification system (20).

### Sleep Apnea Risk Assessment

Questions assessing sleep apnea were taken from the University of Maryland Medical Centre Questionnaire for Sleep Apnea. These were self-rated assessments of the frequency and severity of snoring and pauses in breathing during sleep (a more severe symptom of OSA) over the previous 12 months. To assess the severity of the snoring, the following question was asked: “In the last 12 months, how frequently do you experience or have you been told about snoring loud enough to disturb the sleep of others?” [0 = never; 1 = rarely (less than once a week); 2 = occasionally (1–3 times a week); 3 = frequently (>3 times a week)]. To assess the severity of pauses, the following question was asked: “In the last 12 months, how often have you been told that you have “pauses” in breathing or stop breathing during sleep?” [0 = never; 1 = rarely (less than once a week); 2 = occasionally (1–3 times a week); 3 = frequently (>3 times a week)].

The assessment of the severity of disrupted sleep in the past week was made using Question 13 from the Assessment of Quality of Life (AQoL) Questionnaire-4D (21). The severity of disrupted sleep was rated as 1 = “I am able to sleep without difficulty most of the time”; 2 = “My sleep is interrupted some of the time, but I am usually able to go back to sleep without difficulty”; 3 = “My sleep is interrupted most nights, but I am usually able to go back to sleep without difficulty”; 4 = “I sleep in short bursts only. I am awake most of the night.” Dichotomous categories for sleep measurement were created as follows: snoring (yes = reported snoring, no = no snoring); pauses (yes = reported pauses, no = no pauses); disrupted sleep (yes = at least some of the time, no = rarely).

### Physical Activities and Physical Health

Physical activity in the past 7 days was assessed using the International Physical Activity Questionnaire (IPAQ) short form (22). Electronic scales measuring up to 200 kg were used to assess weight (kilograms); height (metres) was taken against a measure on a wall. Body Mass Index (BMI) was calculated as weight/height^2^. Participants were categorized according to WHO criteria (23) as underweight (BMI < 18.5), normal (BMI 18.5–24.99), overweight (BMI 25–29.99) or obese (BMI ≥ 30). Participants’ absolute 5-year cardiovascular disease risk was determined by Framingham risk equation (24). Participants’ history of diagnosed cardiovascular disease was ascertained by asking participants if they had ever been told by a doctor that they had any of the following: heart attack, angina, stroke/transient ischemic attack (TIA), or other heart disease, e.g., arrhythmias. They were asked to bring all medications to the interview, and those relevant to cardiometabolic conditions were identified. Details of all medication used in the 4 weeks prior to interview was recorded; this was based on self-report or review of medication charts (25).

Participants were also asked to provide a fasting blood sample for analysis of high density lipoprotein cholesterol, triglyceride, and plasma glucose levels. Metabolic syndrome was defined using the harmonized criteria developed by the International Diabetes Federation Task Force on Epidemiology and Prevention and related expert organizations (26). These criteria for metabolic syndrome require three of the following five risk factors to make the diagnosis: abdominal obesity (at-risk waist circumference ≥94 cm for men and ≥80 cm for women); at-risk diastolic and/or systolic blood pressure (systolic blood pressure ≥130 mmHg and/or a diastolic pressure ≥85 mmHg); at-risk levels of fasting blood glucose (≥5.6 mmol/L), triglycerides (≥1.7 mmol/L), or HDL-C (<1.0 mmol/L for men and <1.3 mmol/L for women). Abdominal obesity was defined as a waist circumference ≥94 cm for men and ≥80 cm for women. Hypertension was diagnosed if the person had a systolic blood pressure ≥130 mmHg and/or a diastolic pressure ≥85 mmHg. The thresholds for blood glucose, triglycerides, and lipids were: glucose ≥5.6 mmol/L; triglycerides ≥1.7 mmol/; HDL-C <1.0 mmol/L for men and <1.3 mmol/L for women. People receiving medications for hypertension, hyperlipidemia, or hyperglycemia were considered to meet the relevant criterion.

The IPAQ short form (22) was used to assess the amount of time participants spent in both vigorous and moderate exercise, and the amount of time they spent sitting on a typical weekday, during the last 7 days. The total time spent in various activities over the previous 7 days was classified according to Australian Bureau of Statistics criteria as applied in the National Survey of Mental Health and Wellbeing (27) into four levels of activity: very low, low, moderate, and high.

### Psychotropic Medications

Information about antipsychotics, mood stabilizers, antidepressants, and other sedative medications taken in the 4 weeks prior to the interview was collected. Outpatients were asked to bring their medications to the interview, and for inpatients, the drug charts were reviewed. Antipsychotic medications were sub-classified as typical antipsychotics and atypical antipsychotics.

### Substance Use

Respondents were asked how often they had used alcohol, cannabis, amphetamines, and other drugs in the previous year, and were classified as: 1 = not used; 2 = monthly or less than monthly; 3 = weekly/daily (19). Alcohol dependence and risk categories were measured using the Alcohol Use Disorders Identification Test (AUDIT) (28). Lifetime diagnoses of alcohol, cannabis, and other substance abuse/dependence were assessed using the DIP (19). Caffeine consumption was quantified based on amount per day on average in the previous 4 weeks.

### Diagnosis, Psychopathology, and Cognitive Function Assessment

Diagnostic assessment was based on a semi-structured clinical research interview, the DIP (19). Diagnoses were made according to the ICD-10 classification system (20). Psychiatric symptoms were systematically interrogated using the DIP. Symptoms of hallucinations, delusions, or subjective thought disorder in the past 1 month and symptoms of anxiety, including worry, panic, anxiety, and obsession over the past 12 months were recorded. A brief cognitive assessment tool was employed. This comprised the: (i) National Adult Reading Test (NART) Revised (29) and (ii) Digit-Symbol Coding Test (DSCT) from the RBANS battery (30). Participants’ subjective experience of forgetfulness over the past 12 months prior to the interview was assessed and the results were classified as: 1 = able to remember most things; 2 = somewhat forgetful; 3 = very forgetful.

### Quality of Life Assessment

The quality of life in the past week was assessed using The AQoL-4D 12-item instrument (21). The AQoL-4D independently models all the sub-dimensions of health (independent living, social relationships, physical senses, psychological well-being, and illness) and combines sub-models to obtain a multi-attribute utility score. Scores from the first four dimensions form the multi-attribute utility score. Algorithms for AQoL scoring were obtained from http://www.aqol.com.au/index.php/scoring-algorithms?id=82. Where negatively skewed (AQoL utility scores), data were transformed using the appropriate log transformations.

### Statistical Analyses

Descriptive statistics were reported as means and SDs. Dichotomous categories were created: age (18–34 vs. 35–64); obesity – BMI ≥ 30 (no vs. yes); absolute 5-year cardiovascular disease risk by Framingham risk equation (low vs. high = medium risk or high risk); any formal activity, including paid or unpaid work, or study (no vs. yes); subjective memory forgetfulness (no = able to remember most things vs. yes = somewhat forgetful or very forgetful); Fagerstrom nicotine dependence categories (low = low or very low vs. high = moderate, high, or very high); AUDIT risk (low vs. high = hazardous, harmful, or dependent). Univariate logistic regression was performed to explore associations between and among potential risk factors (independent variables) and the dependent variables (sleep apnea – snore, sleep apnea – pause, and disrupted sleep). Independent samples *t*-test was used to compare the mean scores for the AQoL. A binary logistic regression analysis was conducted to explore potential predictors of OSA symptoms. Variables were divided into four categories (cardiovascular risks; physical activity; social function; and substance abuse) and were entered into the model to estimate multivariate associations, with and without adjusting for age, gender, and BMI. Variables were included in the regression analyses, if they demonstrated significant association with OSA symptoms in the univariate analyses. All statistical analyses were performed with SPSS 22.0. The α value taken to indicate statistical significance was adjusted using Bonferroni correction for multiple comparisons of variables of the same category.

## Results

### Sociodemographic Data

The study sample comprised 1825 participants. Their mean age was 38.4 ± 11.2 years, and 59.6% were male. Most participants were single (61.2%); only 17% were married or in *de facto* relationships. More than half (53.2%) of the participants were not currently engaged in any meaningful activity such as paid or unpaid employment, volunteer job, career, home duty, or study. Most did not engage with any vigorous or moderate physical activity (76.9 and 69.2%, respectively); around half (48.5%) had engaged in any form of physical activity for less than 2 h per week. Nearly all participants (96.7%) had very low to low levels of exercise, according to ABS classification (31). The majority of participants were overweight, with 46.4% being obese (BMI ≥ 30), and the mean BMI was in the obese range (30.5 ± 7.5 SD) (Table 1).

**Table 1:** **Social demographics**.

	*N*	%	Mean ± SD
**Gender**			
Men	1039	59.3	
Women	714	40.7	
**Age**			38.4 ± 11.2
18–34	773	42.4	
35–64	1052	57.6	
**Marital status**			
Single, never married	1117	61.2	
Married/*de facto*	312	17.0	
Separated	376	20.6	
Widowed	20	1.1	
**Education level**			
Complete year 9	410	22.5	
Complete year 10	535	29.3	
Complete year 11	283	15.5	
Complete year 12	574	31.5	
**Physical activity**			
Vigorous (none)	1448	79.6	
Moderate (none)	1255	69.2	
Walking (≤2 h/week)	942	52.3	
Total activity (≤2 h/week)	881	48.5	
Sitting (h/week)			45.7 ± 23.2
**Level of exercise (ABS classification)**			
Very low	611	33.6	
Low	1148	63.1	
Moderate	54	3	
High	6	0.3	
**Meaningful activity (paid or unpaid work or study)**			
Yes	854	46.8	
No	971	53.2	
**Difficulty reading, writing, or both**			
Yes	335	18.4	
No	1490	81.6	
**BMI – criteria from World Health Organization**			30.5 ± 7.5
Underweight	26	1.5	
Normal	409	23.1	
Overweight	516	29.1	
Obese	823	46.4	
**Any history of cardiovascular disease (including hypertension and stroke)**	505	28.5	
**Diagnosis**			
Schizophrenia	857	47.0	
Schizoaffective disorder	293	16.1	
Bipolar disorder with psychotic features	319	17.5	
Depression with psychosis	81	4.4	
Delusional disorder	92	5.0	
Major depression without psychosis	158	8.7	
Screen positive but did not meet full criteria for ICD-10 psychosis	25	1.4	

### Prevalence of Symptoms of Sleep Disturbance

Nearly half of participants reported snoring during sleep (41.9%), while 17.4% reported that they stopped breathing during sleep and 7% reported frequent (>3 times per week) episodes of stopping breathing during sleep. Over half of participants reported disrupted sleep at least some of the night (Table 2).

**Table 2:** **Sleep data of the study population (present state)**.

	*N*	%
**Sleep apnea snoring**		
Never	1018	58.1
Rarely	185	10.6
Occasionally	213	12.2
Frequently	337	19.2
**Sleep apnea (pause)**		
Never	1418	82.6
Rarely	100	5.8
Occasionally	78	4.5
Frequently	121	7.0
**Disrupted sleep**		
Rarely	837	46.3
Some of the night	415	23.0
Most of the night	318	17.6
Short bursts sleep	236	13.1

### The Association between Symptoms of Sleep Disturbance and Sociodemographic Characters

Women reported snoring more frequently than men (*p* < 0.001). Individuals aged 35–64 years had significantly higher odds of snoring (*p* < 0.001), pauses (stopped breathing during sleep) (*p* < 0.05), and disrupted sleep (*p* < 0.05), compared with those aged 18–34 years. People reporting pauses in breathing during sleep were less likely to have any formal work or study activity (*p* < 0.05), be in paid employment in the previous year (*p* < 0.01), or be in paid employment in the previous week (*p* < 0.001) (Table 3).

**Table 3:** **Univariate associations between participant characteristics and symptoms of sleep disturbance**.

	Snore				Pause				Disruptive sleep			
	*N*	%	χ^2^ (*p*)	OR (95% CI)	*N*	%	χ^2^ (*p*)	OR (95% CI)	*N*	%	χ^2^ (*p*)	OR (95% CI)
**Gender**												
Men	399	38.4	13***	1.43 (1.18, 1.73)	117	17.4	0.01	1.01 (0.78, 1.30)	557	51.8	3.6	1.20 (0.99, 1.45)
Women	336	47.1		122	17.5	412			56.4			
**Age**												
18–34	259	34.1	33.5***	1.77 (1.46, 2.16)	110	14.9	5.8*	1.37 (1.06, 1.77)	390	50.7	4.7*	1.23 (1.02, 1.48)
35–64	476	47.9		189	19.3	579	55.8
**Lives alone**												
Yes	208	39.4	2.3	0.85 (0.69, 1.05)	82	15.9	1.2	0.86 (0.65, 1.13)	314	56	1.5	1.13 (0.93, 1.39)
No	514	43.3			210	18.1			637	52.8		
**Formal activity (paid or unpaid work, or study)**												
Yes	345	41.5	0.14	0.97 (0.8, 1.17)	122	15	6.5*	0.72 (0.56, 0.93)	450	52.9	0.39	0.94 (0.78, 1.15)
No	390	42.3			177	19.6		519	54.3			
**Paid employment – past year**												
Yes	232	39.9	1.4	0.88 (0.72, 108)	75	13.2	10.7**	0.62 (0.47, 0.83)	308	51.9	1.2	0.9 (0.74, 1.09)
No	503	42.9			224	19.5		661	54.5			
**Paid employment – past week**												
Yes	156	40.9	0.2	0.95 (0.75, 1.2)	43	11.4	12.1***	0.55 (0.39, 0.77)	194	49.6	3.3	0.81 (0.65, 1.12)
No	579	42.2			256	19.1		775	54.8			

*OR, odds ratio; CI, confidence interval*.

*p < 0.05.

**p < 0.01.

***p < 0.001.

### Associations between Cardiovascular Risks and Symptoms of Sleep Disturbance

People who reported snoring or pause had 1.5–3 times odds than those who did not to meet at-risk criteria for most key cardiometabolic risk factors, including elevated plasma triglycerides and fasting glucose, low HDL cholesterol, and hypertension. Participants reported snoring had odds ratio (OR) of 1.84 [confidence interval (CI) 1.46, 2.32], while those reported pause had OR of 2.26 (CI 1.66, 3.08) to meet criteria for metabolic syndrome and they had a higher 5-year risk of cardiovascular disease [snore OR 1.56 (CI 1.23, 1.98); pause OD 1.65 (CI 1.23, 2.22), respectively]. Snorers were more likely to have reported a history of cardiovascular disease, including angina, heart attack, other heart disease, e.g., arrhythmias, hypertension, and stoke/TIA [OR 2.03 (CI 1.64, 2.52)], as did those who reported pauses in breathing during sleep [OR 2.19 (CI 1.69, 2.84)]. Participants with disrupted sleep were more likely to meet the at-risk criteria for triglyceride levels, but not for other cardiovascular risk factors. Those with disrupted sleep were more likely to have a history of cardiovascular disease [OR 1.47 (CI 1.19, 1.82)] (Table 4).

**Table 4:** **Univariate associations between participant cardiovascular risks and symptoms of sleep disturbance**.

	Snore				Pause				Disruptive sleep			
	*N*	%	χ^2^ (*p*)	OR (95% CI)	*N*	%	χ^2^ (*p*)	OR (95% CI)	*N*	%	χ^2^ (*p*)	OR (95% CI)
**Triglycerides level -criteria from IDF criteria**												
At risk	287	48.2	12.4***	1.5 (1.2, 1.88)	141	24.2	33.4***	2.44 (1.79, 3.33)	350	57.3	6.5	1.33 (1.07, 1.66)
Not at risk	247	38.2			73	11.6			333	50.2
**Fasting plasma glucose level – criteria from IDF criteria**												
At risk	177	49.6	8.9**	1.45 (1.14, 1.86)	76	22	6	1.47 (1.08, 2.02)	205	56	1.2	1.15 (0.9, 1.47)
Not at risk	357	40.3			139	16			478	52.6		
**Blood pressure systolic and diastolic – IDF criteria**												
At risk	406	48.8	30.9***	1.73 (1.43, 2.1)	168	20.7	11***	1.54 (1.19, 1.98)	465	54.5	0.6	1.08 (0.89, 1.3)
Not at risk	310	35.5			125	14.6			474	52.7		
**BMI: overweight – IDF criteria**												
Yes	599	46.5	41.2***	2.16 (1.7, 2.7)	263	20.8	37.5***	3.17 (2.16, 4.66)	701	52.8	0.8	0.91 (0.73, 1.13)
No	122	28.7			32	7.7			240	55.3		
**HDL level – IDF criteria**												
At risk	286	46.4	5.8	1.32 (1.05, 1.66)	130	21.6	13.1***	1.74 (1.29, 2.36)	343	54.5	0.4	1.08 (0.86, 1.34)
Not at risk	244	39.5			82	13.6			336	52.7		
**Metabolic syndrome – IDF criteria**												
At risk	308	50.6	27.4***	1.84 (1.46, 2.32)	140	23.6	27.2***	2.26 (1.66, 3.08)	347	55.6	1.7	1.16 (0.93, 1.45)
Not at risk	218	35.7			72	12			327	52		
**Absolute cardiovascular disease 5-year risk – Framingham risk equation**												
High	205	52	13.4***	1.56 (1.23, 1.98)	92	24	11***	1.65 (1.23, 2.22)	245	60.3	9.5*	1.45 (1.15, 1.84)
Low	361	41			138	16			461	51.2		
**Any history of cardiovascular disease (including hypertension and stroke)**												
Yes	263	54.6	43.2***	2.03 (1.64, 2.52)	124	26.3	35.5***	2.19 (1.69, 2.84)	300	60.4	12.9***	1.47 (1.19, 1.82)
No	459	37.1			170	14.0			644	50.9		

*OR, odds ratio; CI, confidence interval; IDF, International Diabetes Federation; BMI, body mass index; HDL, high density lipoprotein*.

*p < 0.05.

**p < 0.01.

***p < 0.001.

### Associations between Level of Physical Activities, Quality of Life, Psychopathology, and Symptoms of Sleep Disturbance

Participants with pauses in breathing during sleep, but not snoring or interrupted, had higher odds of not to engage in any vigorous activity [OR 1.6 (CI 1.14, 2.26)] or total physical activity [OR 1.53 (CI 1.09, 2.16)]. Participants reporting pauses in breathing during sleep had lower scores on measures of total quality of life (*p* < 0.001), independent living (*p* < 0.05), and psychological wellbeing (*p* < 0.001) when compared with those without pauses (Table 5).

**Table 5:** **Univariate associations between participant physical activities, quality of life, and symptoms of sleep disturbance**.

		Snore				Pause				Disruptive sleep			
		*N*	%	χ^2^ (*p*)	OR (95% CI)	*N*	%	χ^2^ (*p*)	OR (95% CI)	*N*	%	χ^2^ (*p*)	OR (95% CI)
**Vigorous activity (h/week)**													
0		598	43.2	3.8	1.27 (1.0, 1.6)	254	18.7	7.5**	1.6 (1.14, 2.26)	770	53.7	0.0	1.0 (0.8, 1.27)
>0		137	37.5			45	12.6	198		53.5			
**Moderate activity (h/week)**													
0		493	41.2	1.0	0.9 (0.74, 1.11)	207	17.6	0.9	1.03 (0.79, 1.36)	664	53.3	0.2	0.96 (0.78, 1.17)
>0		239	43.7			91	17.1			301	54.4		
**Walk (h/week)**													
≤2		389	43.4	2.0	1.15 (0.95, 1.39)	169	19.3	4.2	1.3 (1.0, 1.68)	510	54.6	0.8	1.1 (0.9, 1.31)
>2		336	40.1			128	15.5		449	52.5			
**Total activity (h/week)**													
0		105	47.7	3.4	1.3 (0.98, 1.73)	50	23.4	6.0*	1.53 (1.09, 2.16)	122	54.2	0.0	1.03 (0.77, 1.36)
>0		630	41.2			249	16.6	846		53.6			

			**Mean**	**SD**			**Mean**	**SD**					

AQoL (log)	No	985	−0.39	0.38	NS	1377	−0.37	0.35	*P* < 0.001				
	Yes	713	−0.40	0.36	NS	285	−0.47	0.41					
Indep living (log)	No	1008	−0.07	0.14	NS	1407	−0.07	0.14	*P* < 0.05				
	Yes	728	−0.08	0.14		293	−0.09	0.15					
Social rel (log)	No	991	−0.26	0.32	NS	1389	−0.25	0.31	NS				
	Yes	718	−0.24	0.29		287	−0.26	0.30					
Physic sense (log)	No	1012	−0.03	0.06	NS	1412	−0.03	0.05	NS				
	Yes	732	−0.03	0.05		296	−0.03	0.06					
Psy well-being (log)	No	1009	−0.09	0.15	NS	1409	−0.09	0.14	*P* = 0.000				
	Yes	730	−0.10	0.14		294	−0.13	0.18					

*OR, odds ratio; CI, confidence interval; AQoL (log), algorithms for the Assessment of Quality of Life-4D multi-attribute utility score; Indep living (log), algorithms for independent living; Social rel (log), algorithms for social relationships; Physic sense (log), algorithms for physical senses; Psy well-being (log), algorithms for psychological well-being*.

*p < 0.05.

**p < 0.01.

***p < 0.001.

Participants with disrupted sleep but not snoring or pauses in breathing during sleep had higher odds of experiencing hallucinations, delusions or subjective thought disorder (*p* < 0.001), depressive symptoms (*p* < 0.001), and manic symptoms (*p* < 0.001) in the past month. Anxiety symptoms were more likely to occur in those whose sleep was characterized by snoring (*p* < 0.01), pauses (*p* < 0.001), and disruption (*p* < 0.001) (Table 6).

**Table 6:** **Univariate associations between subjects’ psychopathology at present state and symptoms of sleep disturbance**.

	Snore				Pause				Disruptive sleep			
	*N*	%	χ^2^ (*p*)	OR (95% CI)	*N*	%	χ^2^ (*p*)	OR (95% CI)	*N*	%	χ^2^ (*p*)	OR (95% CI)
**Hallucinations, delusions, or subjective thought disorder in the past 1 month**												
Yes	404	41.5	0.1	1.02 (0.85, 1.24)	182	19.1	4.4	1.28 (0.99, 1.64)	576	57.4	12.9***	1.41 (1.17, 1.7)
No	331	42.4			117	15.3			393	48.9		
**Any depressive symptoms in the past 1 month**												
Yes	210	41.5	0.1	0.98 (0.79, 1.2)	97	19.8	2.7	1.25 (0.96, 1.64)	366	70.2	81.1***	2.67 (2.15, 3.32)
No	525	42.1			202	16.5			603	46.9		
**Screen for mania over the past 1 month**												
Yes	75	48.4	2.9	1.33 (0.96, 1.85)	23	15.4	0.4	0.85 (0.54, 1.36)	114	72.2	23.8***	2.4 (1.68, 3.45)
No	660	41.3			276	17.6			855	51.9		
**Positive general rating of anxiety or phobia in the past 12 months**												
Yes	466	44.6	7.8**	1.32 (1.09, 1.6)	209	20.4	15.7***	1.71 (1.31, 2.24)	663	61.3	63.8***	2.17 (1.79, 2.63)
No	269	37.9			90	13.0			306	42.2		

*OR, odds ratio; CI, confidence interval*.

*p < 0.05.

**p < 0.01.

***p < 0.001.

### Associations between Psychotropic Medications and Symptoms of Sleep Disturbance

Participants taking atypical antipsychotic medications (including clozapine) had lower odds of reporting disrupted sleep than those not taking atypical antipsychotics (*p* < 0.001, respectively). Atypical antipsychotics were not associated with snoring or pauses in breathing during sleep. Mood stabilizers were associated with increased odds of snoring (*p* < 0.001) and pauses in breathing during sleep (*p* < 0.05), but there was no association with disrupted sleep. Taking an antidepressant was associated with an increased likelihood of snoring (*p* < 0.001) and a reduced likelihood of disrupted sleep (*p* < 0.001), but had no significant association with pauses in breathing during sleep (Table 7).

**Table 7:** **Univariate associations between participants’ psychotropic medications and symptoms of sleep disturbance**.

	Snore				Pause				Disruptive sleep			
	*N*	%	χ^2^ (*p*)	OR (95% CI)	*N*	%	χ^2^ (*p*)	OR (95% CI)	*N*	%	χ^2^ (*p*)	OR (95% CI)
**Typical antipsychotics**												
Yes	156	44.4	1.1	1.14 (0.9, 1.44)	63	18.4	0.3	1.1 (0.8, 1.48)	186	51.7	0.7	0.91 (0.72, 1.14)
No	579	41.3			236	17.2			783	54.1		
**Atypical antipsychotics**												
Yes	526	41.9	0.0	1.0 (0.81, 1.23)	203	16.5	2.3	0.81 (0.62, 1.06)	651	50.5	18.5***	0.63 (0.52, 0.78)
No	209	42.0			96	19.6			318	61.6
**Mood stabilizer**												
Yes	237	50.7	20.3***	1.63 (1.32, 2.02)	92	20	3.9*	1.32 (1.0, 1.73)	251	51.8	1.0	0.9 (0.73, 1.11)
No	498	38.7	207	16.3	718	54.4						
**Antidepressant**												
Yes	307	47.7	11.1***	1.39 (1.15, 1.69)	126	19.7	3.7	1.28 (0.99, 1.65)	405	60.1	17.9***	1.52 (1.25, 1.84)
No	428	38.9	173	16.1	564	49.8		
**Clozapine**												
Yes	118	41.7	0.0	1.0 (0.76, 1.28)	49	17.6	0.0	1.0 (0.72, 1.42)	126	42.6	17.5***	0.59 (0.46, 0.75)
No	617	42.0			250	17.4			843	55.8		

*OR, odds ratio; CI, confidence interval*.

*p < 0.05.

**p < 0.01.

***p < 0.001.

### Associations between Substance Use and Symptoms of Sleep Disturbance

People who were at risk of hazardous/harmful/dependent drinking or who were current smokers were more likely to have pauses in breathing during sleep and disrupted sleep, while those who used cannabis or tranquilizers monthly or more frequently were more likely to have disrupted sleep. Monthly or more frequent amphetamine use was associated with lower incidence of pauses. Caffeine intake of <200 mg/day was associated with a lower incidence of snoring and pauses in breathing during sleep (Table 8).

**Table 8:** **Univariate associations between substance use and symptoms of sleep disturbance**.

	Snore				Pause				Disruptive sleep			
	*N*	%	χ^2^ (*p*)	OR (95% CI)	*N*	%	χ^2^ (*p*)	OR (95% CI)	*N*	%	χ^2^ (*p*)	OR (95% CI)
**Alcohol risk (AUDIT)**												
Low	496	41.7	0.1	1.03 (0.84, 1.26)	185	15.9	5.5*	1.36 (1.05, 1.76)	627	51.1	9.7**	1.37 (1.12, 1.68)
High	239	42.5			114	20.5			342	59		
**Current smoker**												
No	242	42.2	0.0	0.99 (0.81, 1.21)	75	13.2	10.8***	1.6 (1.21, 2.13)	297	49.6	5.6*	1.27 (1.04, 1.54)
Yes	490	42.0			223	19.6			663	55.5		
**Cannabis use – past year**												
<monthly	304	40.6	0.0	1.02 (0.8, 1.3)	136	18.7	0.0	1.0 (0.75, 1.38)	391	51.0	9.8**	1.46 (1.15, 1.86)
≥monthly	176	41.1			79	18.9			264	60.4		
**Amphetamine use – past year**												
<monthly	256	41.6	0.6	0.84 (0.54, 1.31)	130	21.7	6.3*	0.43 (0.22, 0.84)	347	54.8	2.5	1.42 (0.92, 2.2)
≥monthly	36	37.5			10	10.5			62	63.3		
**Tranquilizers use – past year**												
<monthly	77	45.6	2.2	0.6 (0.3, 1.19)	43	26.1	0.0	0.95 (0.44, 2.03)	108	62.1	5.8*	2.58 (1.17, 5.68)
≥monthly	15	33.3			11	25.0			38	80.9		
**Caffeine (mg/day)**												
<200 mg	252	37.3	9.8**	1.37 (1.15, 1.67)	95	14.3	7.4**	1.44 (1.11, 1.88)	372	53.6	0.0	1.0 (0.83, 1.21)
≥200 mg	483	44.8			204	19.4			597	53.7		

*OR, odds ratio; CI, confidence interval; AUDIT, Alcohol Use Disorders Identification Test*.

*p < 0.05.

**p < 0.01.

***p < 0.001.

### Multiple Logistic Regression Models Adjusting for Multiple Confounders

After adjusting for gender, age, and BMI in multiple logistic regression models, the odds of pauses in breathing during sleep in participants with a positive history of cardiovascular disease remained 1.96 times (95% CI, 1.19, 3.48) higher than those without the history of cardiovascular disease. Participants who were at high risk of hazardous/harmful/dependent drinking had odds ratio of snoring 1.59 times (CI 1.07, 2.38) and pause in breathing during sleep 1.882 times (CI 1.15, 3.09) in low risk participants (Table 9).

**Table 9:** **Logistic regression model for the relationship between subjects’ OSA symptoms and disease factors**.

	Snore				Pause			
	Unadjusted OR (95% CI)	*P*	Adjusted OR (95% CI)	*P*	Unadjusted OR (95% CI)	*P*	Adjusted OR (95% CI)	*P*
**Physical health**								
Metabolic syndrome IDF criteria	1.84 (1.46, 2.32)	<0.001	0.67 (0.41, 1.05)	NS	2.26 (1.66, 3.08)	<0.001	0.632 (0.37, 1.09)	NS
Absolute cardiovascular disease 5-year risk equation	1.56 (1.23, 1.98)	<0.001	1.16 (0.67, 2.01)	NS	1.65 (1.23, 2.22)	<0.001	1.325 (0.69, 2.54)	NS
Any history of cardiovascular disease	2.03 (1.64, 2.52)	<0.001	1.40 (0.85, 2.30)	NS	2.19 (1.69, 2.84)	<0.001	1.959 (1.19, 3.48)	*P* < 0.05
**Physical activities**								
Vigorous activity (h/week)	1.27 (1.0, 1.6)	NS	1.22 (0.72, 2.06)	NS	1.6 (1.14, 2.26)	<0.01	0.935 (0.48, 1.83)	NS
Walk (h/week)	1.15 (0.95, 1.39)	NS	1.01 (0.58, 1.75)	NS	1.3 (1.0, 1.68)	<0.5	0.771 (0.39, 1.52)	NS
Total activity (h/week)	1.3 (0.98, 1.73)	NS	0.81 (0.43, 1.50)	NS	1.53 (1.09, 2.16)	<0.5	0.834 (0.39, 1.77)	NS
**Social function**								
Formal activity (paid or unpaid work, or study)	0.97 (0.8, 1.17)	NS	1.38 (0.77, 2.50)	NS	0.72 (0.56, 0.93)	<0.05	1.161 (0.59, 2.30)	NS
Paid employment – past year	0.88 (0.72, 108)	NS	0.97 (0.47, 2.00)	NS	0.62 (0.47, 0.83)	<0.01	0.927 (0.39, 2.19)	NS
Paid employment – past week	0.95 (0.75, 1.2)	NS	0.86 (0.43, 1.71)	NS	0.55 (0.39, 0.77)	<0.001	0.399 (0.16, 1.01)	NS
**Substance abuse**								
Alcohol risk (AUDIT)	1.03 (0.84, 1.26)	NS	1.59 (1.07, 2.38)	*P* < 0.05	1.36 (1.05, 1.76)	<0.05	1.882 (1.15, 3.09)	*P* < 0.05
Current smoker	0.99 (0.81, 1.21)	NS	0.77 (0.43, 1.37)	NS	1.6 (1.21, 2.13)	<0.001	0.746 (0.37, 1.51)	NS
Amphetamine use – past year	0.84 (0.54, 1.31)	NS	1.53 (0.81, 2.89)	NS	0.43 (0.22, 0.84)	<0.05	0.562 (0.22, 1.42)	NS
Caffeine (mg/day)	1.37 (1.15, 1.67)	<0.01	1.60 (1.03, 2.51)	*P* < 0.01	1.44 (1.11, 1.88)	<0.001	1.643 (0.94, 2.89)	NS
**Gender**	1.43 (1.18, 1.73)	<0.001	1.17 (0.76, 1.80)	NS	1.01 (0.78, 1.30)	NS	1.507 (0.89, 2.54)	NS
**Age**	1.77 (1.46, 2.16)	<0.001	2.21 (1.45, 3.39)	*P* < 0.001	1.37 (1.06, 1.77)	<0.05	1.141 (0.68, 1.91)	*P* < 0.05
**BMI**	2.16 (1.7, 2.7)	<0.001	1.21 (0.73, 2.00)	NS	3.17 (2.16, 4.66)	<0.001	2.236 (1.09, 4.58)	*P* < 0.05

*OSA, obstructive sleep apnea; OR, odds ratio; CI, confidence interval; BMI, body mass index*.

## Discussion

In this representative sample of people with psychotic illness, symptoms of OSA were highly prevalent, with rates of pauses in breathing during sleep and snoring 17.4 and 41.9%, respectively. However, these results are likely to be underestimates given that 83% of patients were neither married or in a *de facto* relationship and, thus, likely had no regular bed partner to notice these symptoms during sleep. Our results confirm and add precision to findings from previous studies with small samples of selected patients with schizophrenia (15–18). Further, the prevalence of OSA in our study sample was much higher than the prevalence of OSA in general population, which is estimated to be 3–5% (1, 32–36).

Our study has demonstrated that symptoms of OSA (snoring and pauses in breathing during sleep) were associated with higher likelihood of meeting at-risk criteria for key cardiometabolic risk factors. Symptoms of OSA were associated with a higher rate of a reported history of cardiovascular diseases. The association between pause in breathing during sleep and reported history of cardiovascular disease remained significant after adjusting for BMI, age, and gender. Previous studies in the general population have found that OSA is a significant independent risk factor for CVD-related mortality and a composite endpoint of all-cause mortality and incident stroke (37). Additionally, obesity, male gender, older age, and increased neck circumference are the most significant risk factors for OSA. Thus, it is likely that OSA and obesity may impact on each other to set up a vicious cycle, thereby creating severe cardiometabolic disease.

We found that OSA symptoms, such as snoring and pauses in breathing during sleep, did not impact on the severity of psychopathology, including depressive, psychotic, and manic symptoms, except anxiety symptoms. Disrupted sleep was associated with more severe symptoms in all domains measured. We are not aware of any previous studies examining the associations between OSA and psychopathology in people with psychosis; however, a number of case studies have reported a reduction in positive and negative symptoms of psychosis when Continuous Positive Airway Pressure (CPAP) treatment was initiated (38–41).

Our results revealed an unexpected finding. People prescribed with more obesogenic and sedative atypical antipsychotics were less likely to report subjective sleep disturbances, than those who were prescribed other psychotropic drugs. This contradicts an earlier retrospective study, which found that atypical antipsychotic medications were independently associated with OSA, and that individuals taking atypical antipsychotics had more severe sleep apnea when adjusted for BMI, sex, and use of benzodiazepines and sleeping aids (42). It has been suggested that the sedative actions of atypical antipsychotics may reduce activity of the hypoglossal and recurrent laryngeal nerve on upper airway musculature; however, this is not reflected in our data. The finding that the use of atypical antipsychotics is associated with reduced odds of disrupted sleep could be explained by the hypnotic effects of these medications, which could possibly cause a reduction in reported sleep symptoms. Another explanation for the difference between our results and Rishi et al. (42) is that Rishi compared people taking atypical antipsychotics with participants who were not taking any hypnotic medication, whereas we have compared people with psychotic illnesses on a variety of typical antipsychotics, atypical antipsychotics, mood stabilizers, and benzodiazepines. Interestingly, our study showed that the use of mood stabilizers and antidepressants was associated with symptoms of OSA. As mood stabilizers and antidepressants are commonly used as adjunctive medications in psychosis, this finding suggests that the prescribing of these drugs may increase the risk of OSA. Another possible explanation for this new finding is, people with OSA may be more depressed and fatigued and, therefore, more likely to be prescribed antidepressants and mood stabilizers.

Pauses in breathing during sleep, indicating the more severe form of OSA, were associated with lower rates of employment or study. Evaluation of health-related quality of life showed that our participants reporting more severe form of symptoms of OSA had significant impairments in the domains of independent living and psychological well-being. It is possible that current sleep symptoms may negatively impact on social participation; alternatively, people who are disengaged from social activity may also sleep poorly, perhaps as a result of depression or poor physical health. However, if sleep disorders do cause a reduction in social function, treatment of these disorders could be associated with improved function and productivity. This hypothesis could be tested in clinical trials.

In this study, we found that symptoms of sleep apnea and symptoms of disrupted sleep impact on different clinical domains in people with psychosis. Symptoms of sleep apnea are more likely to have negative relationship with people’s cardiometabolic risk, social participation, and physical activity, while disrupted sleep has stronger association with active psychiatric symptoms. This suggests that the mechanism of the effect of sleep apnea on patients’ clinical outcomes is different from sleep deprivation alone.

In conclusion, this study provides robust evidence that sleep disorders are prevalent in people with psychotic illness and may contribute a further risk factor for cardiovascular disease. The disease-specific determinants of OSA in people with psychosis should be elucidated in future studies. Further investigation and treatment of OSA in people with psychosis may be beneficial in reducing the burden of cardiovascular disease, productivity, and quality of life.

### Limitations

This study had several limitations: The inherent biases of observational data without clear objectives apply to this study. The measures of symptoms of sleep apnea are indirect and self-reported. This study did not specifically examine the polypharmacy interaction or the medication compliance. Due to the nature of cross-sectional study, we can only identify associations in the data.

## Ethics Statement

This study was approved by the appropriate institutional human research ethics committees at each of the study sites, and have therefore been performed in accordance with the ethical standards laid down in the 1964 Declaration of Helsinki and its later amendments. All participants provided written, informed consent prior to participation.

## Author Contributions

DL wrote the first draft of the paper, undertook the initial data analyses, and collated and edited successive versions of the paper. HM has expertise in sleep disorders in people with psychosis and contributed to interpretation of the data and writing of the manuscript. DF has high level statistical skills and has undertaken extensive analyses of the SHIP data and written a number of papers on the physical health data from SHIP. She contributed to the analysis and interpretation of the data and to the writing of the manuscript. GW is an endocrinologist who has been involved with the SHIP project for many years. He contributed to the understanding and interpretation of the data and to the final manuscript. VM, DC, AW, AM, and CG were part of the team that designed the SHIP project and organized the collection of the SHIP dataset. Besides being responsible for the original data, they also contributed to the writing of the paper and understanding of the SHIP data. AM also made additional contributions to the statistical aspects of the paper.

## Conflict of Interest Statement

The authors declare that the research was conducted in the absence of any commercial or financial relationships that could be construed as a potential conflict of interest.
